# Can aerobic exercise really improve factors related to decision-making capacity in aging? What has been measured, what has been assumed, and what needs to change

**DOI:** 10.3389/fspor.2026.1905912

**Published:** 2026-08-26

**Authors:** Jonathan A. Cortés Silva, Christina A. Davis, Diego Lievano Parra, Valeria V. González

**Affiliations:** 1 Max Planck Institute for Multidisciplinary Sciences, Göttingen, Germany; 2 Department of Psychology, Reed College, Portland, OR, United States

**Keywords:** aerobic exercise, cognitive transfer, decision-making, ecological validity, executive function, healthy aging, neural efficiency, prefrontal cortex

## Abstract

As populations age globally, cognitive decline has become one of the most pressing health challenges of our century. Early cognitive decline usually manifests as problems related to decision-making processes. In this context, aerobic exercise has emerged as a promising strategy for preserving and improving brain health. However, a critical question remains underexplored: whether improvements in executive function (EF) tasks truly reflect enhanced decision-making capacity, or whether they index more circumscribed changes in neural efficiency and network specificity. The heterogeneity of protocols has limited efforts to standardize interventions and evaluate their long-term impact. This narrative review examines studies from the last decade that investigated aerobic and combined physical-cognitive training programs in healthy older adults, focusing on their effects on decision-making and associated neural changes. Evidence shows that chronic interventions (6 months to 2 years) improve EF closely related to decision-making, including cognitive flexibility, task switching, and attentional control. These behavioral gains correlate with neural adaptations in the PFC, including improved cerebral oxygenation and preserved white matter microstructure when exercise is sustained over time. At the molecular level, sustained aerobic activity has been associated with increased VEGFR2 and Cathepsin B, and decreased BDNF expression, suggesting systemic plasticity processes that may contribute to cortical integrity. These findings support the potential of long-term, multimodal interventions that integrate physical, cognitive and social components to promote durable neurobiological adaptations and enhance decision-making. Yet, across the reviewed studies, none directly assessed decision-making under ecologically valid conditions, and the improved performance in real-world decision-making remains largely assumed. We argue that the field must move beyond descriptive summaries of cognitive gains and toward a framework that distinguishes between what has been measured, what has been inferred, and what remains to be proven, particularly regarding the generalizability of exercise-induced neural changes to everyday decision-making.

## Highlights

- Aerobic exercise preserves and improves brain health through mechanisms linked to neural efficiency rather than broad cognitive capacity gains.
- Chronic (6 to 24 months) exercise improves executive function, but transfer to real-world decision-making remains assumed, not demonstrated.
- Executive function tasks are routinely used as proxies for decision-making, a conceptual leap that requires critical scrutiny.
- Long-term multimodal programs promote neuroadaptations and executive control, yet their ecological validity for decision-making outcomes remains to be established.

## 1 Introduction

As populations age globally, cognitive decline has become one of the most pressing health challenges of the 21st century (1). Among the various domains of cognition affected by aging, executive functions (EF), which include working memory, cognitive flexibility, inhibitory control, and decision-making, are particularly vulnerable (2–4). These functions are largely supported by the prefrontal cortex (PFC), region known to undergo significant structural and functional alterations with age (5–12).

In recent decades, a growing body of evidence has emphasized the potential of lifestyle-based interventions to promote cognitive health during aging (13). Among these, physical activity, particularly aerobic exercise, has emerged as one of the most robust and accessible strategies for maintaining or even improving executive functioning (14–19). Complementing physical exercise are cognitive training interventions, structured activities aimed to improve specific cognitive domains which have also shown promise; with recent evidence suggests that engaging in both physical and cognitive stimulation may lead to additive or even synergistic effects on brain health (12).

The efficacy and mechanisms of these interventions remain unclear, likely due to differences in population characteristics, intervention design, and outcome measures. For example, some studies report improvements only in select cognitive domains, while others observe broad benefits across attention, memory, and executive control (20, 21). This variability highlights the need for a more nuanced synthesis of findings, particularly regarding the role of exercise type, intensity and duration, the potential benefits of combining modalities; as well as the different tasks used to measure those benefits (22–28). Crucially, this variability also reflects a deeper conceptual problem: the cognitive tasks used to evaluate intervention outcomes were often created in clinical contexts to detect decline, not health; and were not intended to generalized to ecologically relevant situations.

This review aims to provide a comprehensive synthesis and critical evaluation of methods used to evaluate the effects of aerobic exercise interventions, alone or in combination with cognitive training, on decision-making performance and associated outcomes in the prefrontal cortex (PFC). The focus relies on studies involving either human participants or animal models that include molecular, structural, or functional brain measures as well as executive functioning evaluations. Studies were grouped thematically to reflect both the nature of the intervention (aerobic alone vs. dual) and the domain of outcomes (behavioral, neuroanatomical, physiological, molecular).

The review also addresses key moderating factors that may shape the effectiveness of interventions, including whether the exercise was acute or chronic, the type and intensity of training, and characteristics of the study population. To ensure a meaningful comparison of findings, it is crucial to define a priori what is meant by executive function and decision making. Executive functions are commonly understood as a set of higher-order cognitive processes, including working memory, inhibitory control, and cognitive flexibility, that enables goal-directed behavior (29). While decision-making is sometimes listed among executive functions, it is more precisely understood as a distinct but dependent construct: it relies on EF component processes but additionally requires the integration of value, risk, and contextual information to guide action selection (30, 31). In other words, EF processes are necessary but not sufficient conditions for effective decision-making. Decision making refers specifically to the ability to evaluate options, anticipate consequences, and select appropriate actions based on internal goals and external feedback (30). This hierarchical relationship is central to the interpretive framework of this review: improvements in EF tasks provide evidence that component processes supporting decision-making have been enhanced, but do not constitute direct evidence of improved decision-making capacity. Ultimately, we aim for this review to function as a conceptual reappraisal that clarifies what has been measured, what has been assumed, and what the field needs to establish in order to make meaningful claims about aerobic exercise and decision-making in healthy aging.

## 2 Materials and methods

This narrative review explores the relationship between aerobic exercise, Prefrontal Cortex (PFC) or Anterior Cingulate Cortex (ACC) function, and decision-making in healthy aging (See Table 1). We conducted a focused search of peer-reviewed scientific literature published between 2015 and 2025. Searches were performed in PubMed, Web of Science, Scopus, and Google Scholar, using combinations of keywords such as “aerobic exercise”, “decision-making”, “healthy aging”, “anterior cingulate cortex”, “executive function”, and “BDNF” contained in the title (Google Scholar) or title and abstract (all other search engines).

**Table 1:** Keyword combinations used across all databases in literature search.

#	Combination
1	Decision making OR Choice AND Healthy Aging AND Prefrontal cortex
2	Healthy Aging OR Brain health AND Prefrontal cortex
3	Healthy Aging OR Brain health AND Prefrontal cortex AND Exercise
4	Healthy Aging OR Brain health AND Prefrontal cortex AND Physical conditioning
5	Decision making AND healthy aging AND prefrontal cortex AND exercise
6	Delay discounting AND Aging AND Prefrontal cortex
7	Executive function AND Older adults AND Prefrontal cortex AND Physical training
8	Executive function AND Healthy aging AND Anterior cingulate cortex AND Aerobic exercise
9	Prefrontal cortex AND Healthy aging AND Physical activity
10	Decision making AND Uncertainty AND Aging AND Prefrontal cortex
11	Delay discounting AND Cognitive control AND Aging

Articles were identified through a multi-step process combining keyword-based database searches and manual screening of reference lists from selected papers. The initial search yielded a total of 492 entries. A first-level screening was conducted to remove duplicates, resulting in 386 entries. Subsequently, broader exclusion criteria were applied, such as topic irrelevance or lack of experimental data, narrowing the pool to 31 studies. The selected articles focused on original research involving either human participants or animal models, and included studies examining aerobic exercise interventions in relation to decision-making performance and PFC/ACC-related outcomes (structural, functional, or molecular). Given that not a single study focuses on decision-making in aging, we use different aspects of cognition as part of the decision-making process (31, 32). Thus, studies that only included a single test or a neuropsychological screening battery were excluded. This decision reflects a core assumption of the present review: that decision-making, as a multidimensional construct, cannot be adequately captured by a single cognitive measure.

**Figure 1:**
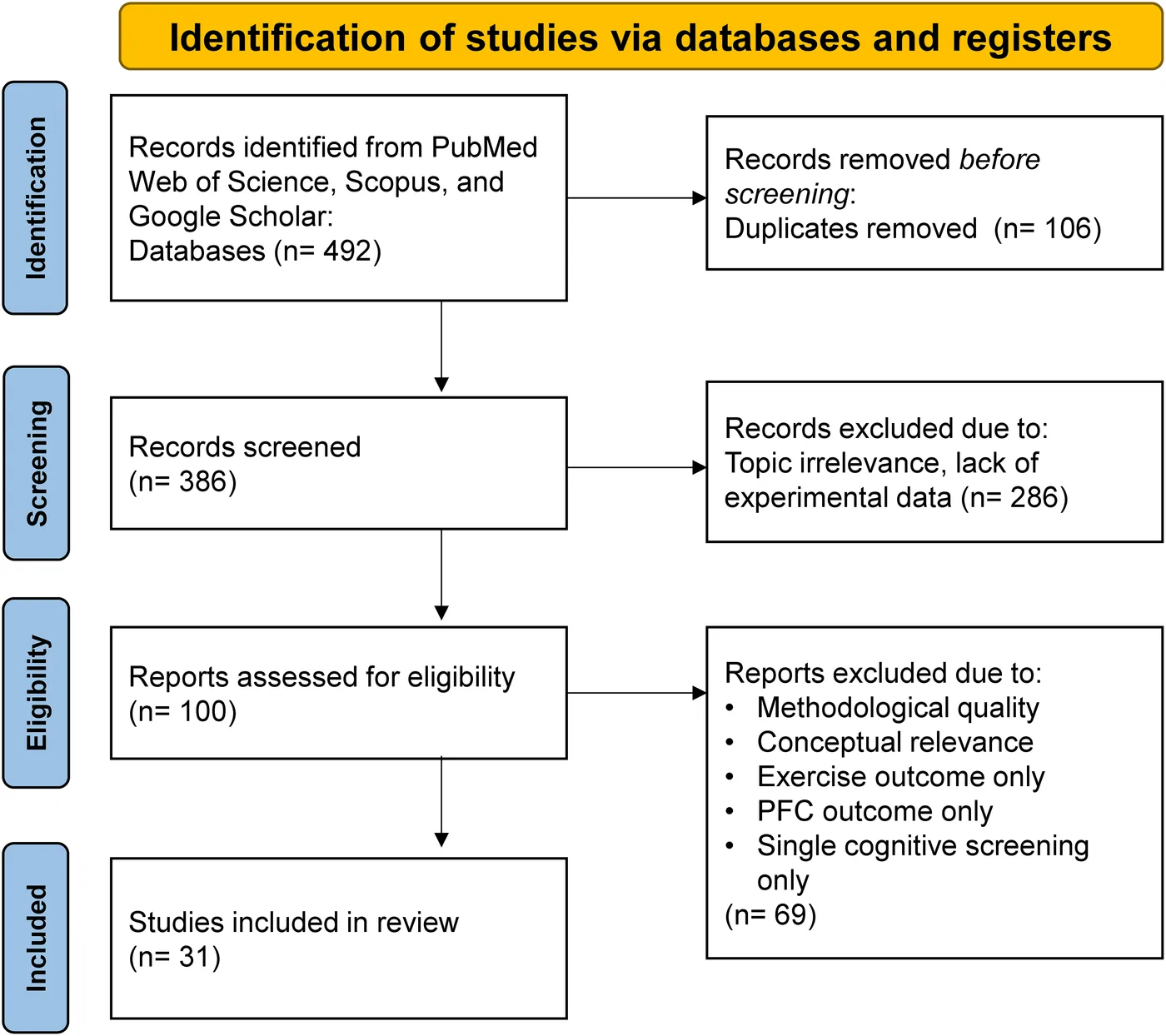
Workflow of articles search and exclusion process. Adapted from Page et al. (82). Licensed under CC BY 4.0.

For each of the 31 included studies, we extracted data on study design, population or model characteristics, intervention parameters (e.g., type, frequency, intensity, and duration of aerobic exercise), and outcomes related to decision-making and PFC/ACC function. The results were written based on 24 of the 31 articles, given that seven of them corresponded to review articles. Those included studies were conducted across different regions, with the majority originating from North America (*n* = 12), followed by Europe (*n* = 7) and East Asia (*n* = 5). Reported effects were captured as described in the original publications, including behavioral metrics, neuroimaging findings, and molecular readouts such as BDNF expression. When applicable, the presence of randomized controlled trial (RCT) designs was noted, along with key methodological strengths and limitations (see Figure 1 for workflow).

## 3 Results

### 3.1 Aerobic effects of exercise intervention on cognitive outcomes in healthy populations

Across the selected studies using aerobic interventions in healthy middle age to older adults (40–99), showed consistent cognitive improvements, particularly in executive function (EF). The majority of the studies had a sample of adults between 55 and 99 years old with two outlier studies, where participants range from 45 to 70 years old and another study focusing on a vital time frame (40–64) for midlife women experiencing menopause. The studies included various forms of aerobic exercise such as brisk walking, rhythmic walking, jogging, stationary cycling, treadmill, muscle training, Tai Chi Chuan (TCC), exergame training, calisthenics, with some happening in individualized sessions while other interventions were carried out in a group setting. The duration of the intervention varied across articles, with 6 studies being acute exercise interventions (1 session to 8-week duration), 3 studies having a subacute intervention (∼12 weeks) and 8 studies having a chronic intervention (∼6 months to 2 years). Across the variety of study designs, aerobic exercise has been shown to influence specific cognitive domains, most notably executive functioning, episodic and verbal memory, and attentional control, all of which contribute to complex decision making.

#### 3.1.1 Executive function and cognitive flexibility

One of the most consistent reported findings across aerobic exercise interventions is improvement in executive function, cognitive flexibility and task switching. In a 6-month randomized control trial, participants who participate in brisk walking for 150 min per week demonstrated significantly enhanced performance on the D-KEFS Trail making test and Letter Fluency compared to their own baseline and compared to the low intensity movement control group (33). This improvement was also correlated with dlPFC morphological integrity, suggesting that key components of EF such as set shifting and verbal fluency, are mediated by the dlPFC and are essential for adaptive decision making. Along the same line, 6-month intervention doing either walking, jogging, cycling or using a cross trainer improved EF measured by combined scores of an operation span task, flanker task and WAIS-R digit span backward task with the improvement positively associated, but not reaching statistical significance, with change in dlPFC thickness (17). Similarly, 6-month intervention of stationary cycling improved executive function and concept formation measured by the Cart Sorting Test and Color-Word Inference Test from the Delis-Kaplan Executive Function system battery. This change was negatively associated with a change in cerebrovascular resistance index, meaning there was better cerebral blood flow and those who had less resistance post-intervention showed greater improvement in executive function (34). This trend was also found in 8 week long aerobic intervention, with improvements in EF measured by time and accuracy during the Trail making test, Executive Control task and Stroop word-color task. This improvement was correlated with induced modulations in PFC oxygenation, implicating that improvements in executive function can be observed relatively quickly with consistent aerobic engagement (35).

On a larger scale, a 2-year aerobic exercise intervention using mild intensity calisthenics showed significant improvement in attentional shift within the 2 years and 6 months post-intervention, supporting the role of aerobic exercise in enhancing cognitive flexibility (36). Similarly, in a study looking at physically active vs. sedentary midlife women, they observed a trend of better performance in the physically active group in the implementation phase of a goal-directed task, which involved applying previously learned associations between specific objects and correct responses to guide their decisions (37). Animal models reinforce these findings as well, a study with rodents subjected to voluntary running in a wheel exhibited superior performance on prefrontal-dependent tasks including attentional set-shifting task and reversal learning, as supported by fewer errors and fewer trials to reach criterion compared to sedentary controls (38). These results suggest that aerobic exercise not only supports cognitive flexibility but also enhances underlying executive neural circuitry. When aerobic exercise is combined with resistance exercise for an acute intervention, EF, measured by Stroop, more-odd shifting and 2-back tests, was improved compared to baseline (39).

#### 3.1.2 Episodic and verbal memory

Aerobic exercise also appears to selectively enhance memory function, particularly in immediate and delayed recall tasks. In a study comparing aerobic exercise and cognitive training, participants in the intervention group showed significant improvements in immediate and delayed memory recall across training sessions measured by WMS-II Logical Memory subtest, outperforming the cognitive training group in these domains (15). Further support for aerobic exercise influence on memory comes from cardiorespiratory (CRF) studies. Higher CRF levels have been associated with greater accuracy in face-name associative memory tasks, with higher for older adults having greater face-name accuracy than their lower CRF peers, suggesting that aerobic fitness provides protection against age-related memory decline (40). While episodic and verbal memory are not typically classified as executive functions, they constitute critical inputs to the decision-making process, particularly in contexts that require retrieving past experiences, evaluating outcomes of prior choices, or updating beliefs based on new information (30). In this sense, aerobic exercise-induced memory improvements may support decision-making by preserving the informational substrate on which evaluative and deliberative processes depend.

#### 3.1.3 Visuoconstruction, attention and processing

Chronic aerobic exercise paired with music improved visuospatial functioning compared to the control measured by a visuospatial constructional ability task where participants were shown different shape figures and were asked to draw them (41). Chronic aerobic exercise in the form of an exergame (e.g., a video game that requires physical movement to play such as Just Dance) was found to decrease the reaction time for working memory, divided attention, go/no go and set shifting tasks, indicating broad attentional enhancement (8). These findings reinforce the view that aerobic interventions may not only reduce cognitive decline but also improve spatial perception/navigation and perceptual-motor integration. Acute aerobic exercise also shows similar results, with intervention improving the rate of correct responses to a refresh function test, where participants view a stimulus, have to remember it during a delay and respond based on it, paired with an observed increase in cerebral blood perfusion in the FPA, OFC and Broca region (39). Participants that self-reported consistently practicing aerobic exercise for a minimum of one month were found to have better processing speed measured by WAIS-IV Coding and Symbol Search tasks, but only when participants did aerobic exercise as well as resistance training, as participants who just did aerobic exercise did not perform better than the sedentary control group (21). However, those results are limited since participants were grouped by self-reported exercise activity in the month prior to the cognitive tests. More broadly, the heterogeneity of tasks used across studies in this section, ranging from visuospatial drawing tasks to reaction time, reflects the absence of a unifying cognitive framework, making it difficult to draw clear conclusions about which specific processes are being modified by aerobic exercise.

#### 3.1.4 Global benefits

In addition to domain-specific gains, several of these studies suggest that aerobic exercise may promote a more generalized cognitive preservation. Six-month intervention of aerobic exercise was found to improve a general “cognitive score”, which comprises episodic memory, processing speed, updating and executive function tasks (17). The overall evidence suggests that aerobic exercise might support both domain-specific and general cognitive health in aging. Variability in intervention length, intensity, and participant risk profile likely modulates the magnitude of the observed effects (see Section 3.5).

However, the notion of “global cognitive benefits” warrants careful scrutiny. Composite cognitive scores, while useful for capturing broad trends, aggregate performance across tasks that may rely on distinct neural systems and that carry different theoretical weights. An improvement in a composite score therefore does not necessarily imply that all contributing domains improved equally. Furthermore, global scores may mask compensatory patterns, where gains in some domains offset losses or stagnation in others, producing an artificially optimistic picture of intervention efficacy.

### 3.2 Neurovascular and neuroplastic changes induced by aerobic exercise

Recent literature points to aerobic exercise inducing neurovascular and molecular adaptations that likely underlie these cognitive changes and gains described in the previous section. In that sense, some studies report reduced cerebral oxygenation in the PFC during cognitive tasks (Stroop test with naming, inhibition and flexibility) following acute aerobic exercise (1–2 sessions), suggesting a neural efficiency effect where the brain requires less metabolic support to maintain or enhance performance (42). Another study using a subacute exergame intervention, found a similar reduction in PFC HbO2 activity during participants' preferred walking speed and fast walking speed, with this reduction being correlated with improved executive functions measured by the Trail-Making test (35). These observed reductions in oxygenation in the PFC during acceleration and sustainment of walking may free up cognitive resources to other processes while walking, improving mobility and fall prevention in the elderly. Similarly, in subacute aerobic exercise interventions, improvements in inhibitory function were accompanied by enhanced baroreflex sensitivity, a physiological marker of autonomic regulation. Additionally, there was a decrease in oxyhemoglobin concentration during the Trails Making Test (7). This relationship suggests that cognitive and cardiovascular adaptations may work in synergy. Memory improvements, particularly in immediate logical memory following 12 weeks of treadmill or stationary cycling, correlate with increased resting cerebral blood flow (CBF) bilaterally in the hippocampi, suggesting a selective rather than global change (15). Overall, while acute aerobic exercise may promote neural efficiency by reducing the metabolic cost of cognition, longer sustained aerobic exercise leads to region-specific increases in resting cerebral perfusion. However, reduced PFC HbO2 is not uniformly interpretable as a marker of neural efficiency. The direction of HbO2 change depends on multiple factors including task demand, exercise intensity, timing of measurement relative to exercise, and individual fitness level. In some contexts, increased HbO2 reflects greater neural recruitment and positive adaptation. The neural efficiency interpretation is therefore most applicable to conditions where performance is maintained or improved alongside reduced oxygenation, a pattern observed in some but not all reviewed studies. However, this pattern is more consistent with an efficiency account than a capacity account of cognitive improvement. That is, aerobic exercise appears to optimize the deployment of existing neural resources rather than expanding the overall cognitive repertoire available to the individual.

At the cellular level, intermittent aerobic exercise for ∼6 months was found to increase the circulation of vascular endothelial growth factor receptor 2 (VEGFR2KDR), a biomarker implicated in brain aging that is essential for angiogenesis (i.e., formation of new blood vessels) and vasculogenesis (i.e., blood vessel development) (33). ∼6-month aerobic exercise intervention for middle-aged and older adults at risk for Alzheimer's found increased levels of plasma Cathepsin B (CTSB), which was correlated with improvements in verbal learning and memory measured by the California Verbal Learning Test. They also found decreased levels of BDNF, which were closely linked to metabolomic alterations (43). In comparison, 10-week aerobic intervention in the form of progressive strength training found an increase in BDNF which was associated with decreased reaction time during the Flanker task but similarly found an increase in CTSB mRNA expression (44). This inconsistency in BDNF findings may be due to differences in the length of intervention or population, as Gaitán et al. (43) used a cohort of participants with risk of Alzheimer's with a 6-month intervention while Kim et al. (44) used healthy participants in a 10-week intervention. Authors also mentioned that acute interventions typically lead to increases in BDNF levels, while chronic intervention effects on BDNF levels are less clear, as well as the possibility of sex differences being a factor. In rodents, 12-day wheel-running intervention was found to have an increment in astrocyte cell body area in the mPFC, OFC and hippocampus as well as an increase in dendritic spine length and density of pyramidal neurons in the mPFC, indicating enhanced synaptic plasticity (38). These molecular findings were in conjunction with an enhancement in cognitive flexibility, highlighting exercise-induced cellular plasticity. Aerobic exercise at the acute level appears to increase brain perfusion and neurotrophin expression (e.g., BDNF), and when exercise is sustained, these mechanisms can trigger a cascade that includes hippocampal neurogenesis, synaptogenesis and enhanced brain structure down the line, especially when paired with cognitive engagement (45). The described changes might reflect both acute and chronic adaptive mechanisms. Indeed, the authors proposed that age-related brain atrophy in healthy aging may be an energy-saving adaptation when faced with reduced physical and cognitive stimulation, and not a pathological loss.

The molecular evidence reviewed here, while compelling, presents its own interpretive challenges. The inconsistency in BDNF findings across studies underscores the difficulty of drawing straightforward conclusions about the molecular pathways linking exercise to cognition. This gap between molecular readouts and cognitive outcomes represents one of the most pressing challenges for the field going forward.

### 3.3 Brain volume, functional brain activation and connectivity patterns during aging and AE intervention

While molecular and neurovascular changes in response to aerobic exercise seem to be relatively fast, frontal cortical volume changes seem to occur with long-term interventions. Jonasson et al. (17) found that baseline dlPFC thickness was positively associated with higher aerobic fitness, measured by VO2 peak. Despite the improvement in cognition after a 6-month intervention, aerobic fitness did not predict dlPFC change but instead predicted hippocampus volume increase. Similarly, in another 6 months intervention there was no significant change in white matter tract microstructure, which reflects the integrity and organization of white matter tracts (46). This may suggest that longer intervention (>6 months) may be required to observe changes in frontal cortex volume or connectivity. In support of this, a 2 year exercise intervention (daily calisthenics and monthly club-based aerobic exercise) was found to preserve bilateral prefrontal volume (bilateral middle frontal gyri) from pre-intervention, whereas the volume of this area decreased consistently over time in the control group. However, the PFC volume preservation faded 6 months after the intervention, suggesting mild intensity exercise is needed to maintain and prevent age-related volume reduction in the prefrontal cortex (36). In spite of this result, it seems that other brain regions are found to be more susceptible to brain volume changes following aerobic exercise that are maintained over time: older adults at high risk for mild cognitive impairments who completed a 12-week resistance training program led to increase in cortical thickness of the right parahippocampal gyrus and right entorhinal gyrus, which was associated with faster response times on an executive control task (Go/No go task), suggesting structural plasticity in regions vulnerable to early cognitive decline (11). These structural findings reveal an important tension: while aerobic exercise can preserve or modestly increase PFC volume under certain conditions, these changes are neither robust nor durable without continued exercise. Volume preservation in the PFC is frequently interpreted as evidence of preserved cognitive function, but this inference assumes a straightforward structure-function relationship that is not always supported empirically. A preserved or enlarged PFC does not necessarily translate into improved decision-making unless the functional organization of the relevant networks is also maintained or enhanced.

Finally, one fMRI study shows that high-fit older adults (55–74 years old) exhibit brain activation patterns more similar to young adults (18–31 years old) than low fit older adults in the posterior and prefrontal cortex, and the thalamus, suggesting that cardiovascular health reduces age-related differences in some brain regions (40). This pattern was also observed when comparing physically active vs. sedentary women, where sustained aerobic exercise showed a trend of differential inferior frontal gyrus activation and greater connectivity with the dlPFC, suggesting network features could act as biomarkers of sustained physical activity and goal-directed behavior maintenance (37). More fit older adults had higher global efficiency, reflecting enhanced functional connectivity across regions, with this pattern being associated with better executive function performance. This seemed to represent a compensatory mechanism, as they found that this came at a cost of lower local efficiency, indicating decreased functional specialization within localized brain networks. Despite this trade-off, authors concluded that physical fitness may support cognitive aging by promoting greater overall network integration, thereby mitigating the effects of age-related neural dedifferentiation (47). Aerobic exercise was also associated with increased cerebral blood perfusion in the frontal pursuit area (PFA) and orbitofrontal cortex (OFC) during a refresh function task. This suggests a potential synergistic mechanism, given the activation levels in the FPA and OFC and the improvement in refresh function following aerobic exercise (39). Participants practicing Tai Chi Chuan (TCC), a Chinese mind-body martial art, showed faster reaction times and increased oxygenated hemoglobin levels in the PFC during an incongruent flankers task (48). These results align with findings of enhanced activation in the ACC and dACC following aerobic exercise (15, 49).

The functional connectivity findings reviewed here are among the most theoretically relevant for decision-making, given that the PFC and ACC are central nodes in the neural circuits underlying value-based choice and cognitive control (30). However, two important caveats apply. First, the compensatory trade-off between global and local efficiency observed by Kawagoe et al. (47) suggests that exercise-induced network reorganization is not uniformly beneficial, enhanced integration across regions may come at the cost of reduced specialization, with uncertain consequences for complex, context-dependent decisions. Second, activation patterns observed during laboratory tasks may not generalize to the distributed, dynamic network coordination required for real-world decisions.

### 3.4 Effects of dual intervention

The importance of active engagement during interventions to achieve optimal cognitive benefits has been highlighted in recent research. Acute interventions involving cycling or cognitive training have been shown to improve executive functions, particularly inhibition and cognitive flexibility, while also reducing cerebral oxygenation in the left and right prefrontal cortex during Stroop test blocks (naming, inhibition, and flexibility block). However, when interventions were combined, that is simultaneous physical and cognitive interventions, an additive effect on cognitive flexibility was reported, suggesting that dual interventions provide greater benefits than either intervention alone, and may represent a more efficient strategy for supporting cognitive health in older adults (42). Similarly, in a subacute intervention (e.g., 12 weeks) all the intervene groups (exercise, cognitive training and dual intervention) showed improvements in executive function, including working memory and cognitive flexibility, but the dual training specifically led to greater improvements in inhibition cost and baroreflex sensitivity compared to single interventions, suggesting synergistic effects (7). Contrarily, subacute interventions of either aerobic exercise (e.g., treadmill or cycling) or cognitive training (named: Strategic memory advanced reasoning training) were found to have differential effects, with the cognitive training group having improvements in tasks related to executive function and the aerobic exercise group having improvements in immediate logical memory (15). This suggests that while there are distinct benefits from each training, a combined approach may therefore not only sustain engagement with the intervention but produce more comprehensive cognitive gains.

Exergame training, which combined cognitive motor training as a game-based physical exercise with music over 8–10 weeks, was found to improve EF in four different tasks: working memory, divided attention (auditory and visual), go/no-go and set-shifting; whereas the balance training control group only improved in set-shifting. Additionally, exergame training reduced theta rhythmic power to auditory stimuli, contrasting the age-related increase typically observed, suggesting a mitigation of cortical changes related to aging, although this effect is not consistently reported in other studies (8). Exergame also uniquely improved motor function, as seen with improvements in gait speed and reduction in PFC oxygenation during walking (8, 35). These motor-specific gains point to the potential of coordination-based exercise interventions to address and prevent mobility deficits in older adults, especially those at risk of impaired gait and falls. Similarly, pairing aerobic exercise with music and dance over a one-year period improved visuospatial functioning beyond that seen with exercise alone or no exercise. While volumes of the right anterior cingulate gyrus, left superior temporal gyrus, and insula were preserved with exercise, the control group had those areas reduced, and only the exercise + music group had an increase in the right superior frontal gyrus (41). This suggests that exercise paired with music, similar to the exergame training, induced greater effects compared to exercise alone or no exercise and therefore can be a great addition to interventions focused on delaying age-related cognitive decline. Finally, in a study comparing dual-task interventions (exercise + mindfulness) to each component alone, all groups exhibited cognitive improvements over time. However, the lack of a strong negative control limits the interpretation of these results (50). Together, these findings support the value of multimodal interventions, especially those combining physical, cognitive and sensory engagement for promoting cognitive health in aging populations.

The dual intervention literature presents a conceptually important but interpretively complex picture. The additive and synergistic effects reported across studies are promising, and the simultaneous engagement of cardiovascular and cognitive systems provides a theoretically compelling mechanism for broader neural adaptation. However, several critical issues limit the conclusions that can be drawn with respect to decision-making specifically. First, the cognitive tasks used to assess dual intervention outcomes, Stroop, Trail Making, go/no-go, working memory updating, remain proxies for high-order cognitive processes. Second, the notion of “synergy” between physical and cognitive training is invoked frequently but rarely operationalized precisely: it is unclear whether the observed additive effects reflect genuine capacity expansion, more efficient neural recruitment, or simply greater task familiarity due to increased cognitive engagement during training. Third, the differential effects observed between aerobic and cognitive training modalities suggest that these interventions may target partially distinct neural systems, raising the question of whether their combination truly integrates these systems or merely produces parallel improvements. Addressing these questions will require intervention designs that include ecologically valid assessments to detect interaction effects between training modalities.

### 3.5 Factors modulating the effects of exercise

#### 3.5.1 Exercise intensity, duration, and type

Exercise intensity was a key modulator of cognitive enhancement. For the purpose of categorized exercise in this review, intensity levels were determined based on the designations provided by the original study authors. In cases where explicit classification was not present, intensity was grouped by physiological load measures (e.g., %VO2max). If those measures were not available, then it was classified by perceived exertion, taking into account the context of older adult populations and their baseline fitness. It is important to note that perceived exercise intensity is relative to an individual's baseline fitness level and age. For example, brisk walking or cycling may be classified as low-intensity aerobic exercise for young adults but can represent moderate or high intensity for older adults, especially those who are sedentary. Low-intensity exercise interventions such as Tai Chi, calisthenics (e.g., furi-furi-guppa) and some of the non-aerobic exercise control interventions such as balance training, yoga or stretching, yielded modest improvements in attention, processing speed and certain aspects of executive function such as inhibitory control and set shifting in older adults (33, 36, 48, 51). These interventions were also associated with preservation of prefrontal cortical volume and increase in blood oxygenation in the PFC over time, although results varied across studies, with some showing only trends towards significance rather than statistically robust effects (33, 36, 48). For moderate-intensity aerobic exercise interventions, such as brisk walking, treadmill, or cycling consistently improved executive function, inhibition, cognitive flexibility, and were associated with increased CBF and change in HbO2 concentration in prefrontal regions and an increase in gray matter volume in frontal and temporal regions (15, 39, 41, 42). High intensity or multimodal training, such as exergame training, cycling with incremental increases in load, resistance training, or Integrated Concurrent Exercise (Aerobic exercise + resistance training) produced broader improvements in executive function, sensory processing, and motor performance with increases in cortical thickness in the PFC, parahippocampal gyrus and entorhinal gyrus, notably applying also to populations at risk for cognitive decline (8, 11, 17, 49). In terms of exercise duration, aerobic exercise benefits on EF and the brain morphology are mainly observed after chronic interventions (>6 months), with slow cortical thickness changes and cognitive gains (8, 17, 33, 36, 37, 40, 41, 45, 50, 51). These effects were found to fade after the intervention, suggesting that effects can reverse if exercise is not sustained over time. Acute effects are less consistent, with the majority of changes seen on the cellular level or regarding brain perfusion (11, 15, 38, 39, 42, 45, 48, 49).

Exercise type also influenced the nature and magnitude of cognitive and neural outcomes. Classic aerobic exercise (e.g., running, cycling, jogging, etc.) significantly enhanced a wider range of cognitive domains. It had a broad improvement in multiple components of executive function such as verbal fluency, set-shifting, refresh function, cognitive flexibility, inhibition, latency of response and response time, as well as higher composite cognitive scores incorporating episodic memory, processing speed, and working memory updating. Non-executive function improvements included better visuospatial processing, working memory, logic (immediate and delayed) and object memory, face-name associative encoding. Neurobiologically, aerobic training was linked to increased white matter volume, increased hippocampal and dlPFC thickness, elevated resetting and task-related CBF in frontal and hippocampal regions, and cellular enhancement like enlarged astrocytic bodies and greater dendritic spine density in the hippocampus, mPFC and OFC, along with preservation of widespread cortical and subcortical structures (15, 17, 33, 38–42, 49). In contrast, resistance training enhanced a smaller subset of executive functions including inhibition, conversion functions, and reaction time, and was associated with activation of the dlPFC and FPA and a nonsignificant trend towards increased cortical thickness in the right entorhinal cortex (11, 39). Combined interventions (AE + resistance training) yielded similar cognitive improvements, including gains in inhibition and conversion functions and activated more brain regions than aerobic exercise or resistance training alone by having synchronous activation of blood flow in the dlPFC, FPA, and OFC suggesting a synergistic mechanism (39). Combined intervention was also found to perform significantly better on WAIS-IV Symbol Search, Letter Fluency, and Stroop Color-Word compared to the cardio group, further supporting an enhanced effect (21). Mind-body interventions such as Tai Chi or mindfulness-based stress reduction (MBSR) combined with aerobic exercise improved inhibitory control and had an increase in oxy-Hb in PFC but not episodic memory and EF (48, 50). Finally, cognitively enriched exercises, including exergames and dual task aerobic exercise, produced improvements on motor performance, working memory, divided attention (auditory and visual) set shifting flexibility and inhibition (8, 42).

In summary, exercise interventions produced domain-specific cognitive and neural benefits with outcomes depending on a constellation of parameters. Synthesizing across all modalities, the most robust cognitive gains, particularly in executive function, memory and processing speed were observed with moderate to high intensity exercise when sustained over longer durations and further enhanced when interventions were cognitively engaging. These patterns are discussed further in relation to transfer effects and ecological validity in Discussion.

#### 3.5.2 Tests used in studies vs. skill evaluated

Across the reviewed studies, variability was observed in both the selection of cognitive tests and the interpretations of the cognitive domains they assessed. This may have influenced outcomes related to exercise-induced cognitive changes. For instance, the Stroop test was most frequently used to evaluate executive function. While one study incorporated the Stroop as a broader cognitive battery to compute a composite EF score (50), others utilized the task to target more specific subcomponents such as inhibition and cognitive flexibility (42). In contrast, another study, though also categorizing it under EF, focused specifically on Stroop interference effects, which are more tied to attention and reaction time (39). Similarly, the Flanker task was used to measure EF (17) but some studies specified measuring inhibition and/or attention with the same test (48, 50). The Trail making test was interpreted variably, where in some studies it was used to assess speeded set-shifting as a measure of EF (33) while another study used it to calculate a processing speed composite score (17). The Go/No-Go Task was used to broadly measure EF and inhibition (11), while in other studies it was specifically used to measure inhibition and attentional performance (8).

This variability in task interpretation is not merely a methodological inconvenience; it reflects a deeper conceptual problem that runs through the entire literature. When the same task is used to operationalize different constructs across studies, cumulative evidence becomes difficult to synthesize and potentially misleading. More critically, none of the tasks reviewed here, Stroop, Flanker, Trail Making, Go/No-Go, were designed to assess healthy aging. They were developed primarily as clinical screening tools or laboratory measures of specific cognitive processes, and their ecological validity for capturing the kind of value-based, consequence-sensitive, goal-directed choices that characterize real-world decisions is limited (30, 31).

## 4 Discussion

Aerobic exercise benefits cognition in aging, with particularly robust effects on executive functions such as cognitive flexibility, inhibition, and working memory. These cognitive gains are accompanied by neurobiological changes in brain regions like the prefrontal cortex (PFC) and anterior cingulate cortex (ACC). Improvements include enhanced structural integrity, functional connectivity, cerebral perfusion, and molecular adaptations such as increased BDNF expression and reduced neuroinflammation At the molecular level, aerobic exercise engages signaling pathways that reduce oxidative stress, modulate the HPA axis, and improve neural metabolism and synaptic plasticity (38, 52–54). This biological readiness likely enhances the brain's responsiveness to additional cognitive stimulation (Table 2).

**Table 2:** Summary of results by intervention type, brain adaptation, cognitive benefit and key distinction as reported in the studies reviewed.

Intervention type	Example	Brain adaption	Predominant cognitive benefit	Key distinction
Acute	>6 month intervention	↑ CBF, ↑ HbO_2_, ↑ BDNF, transient PFC activation	Inhibition, attention, processing speed	Rapid physiological effects without structural change
Chronic	<6 month intervention	Hippocampal & PFC plasticity, improved functional connectivity and cerebrovascular function	Executive function and episodic memory	Long-lasting neuroplastic adaptations
Low intensity	Tai Chi	PFC oxygenation, preserved frontal volume	Attention, set-shifting	Accessible intervention with modest cognitive benefits
Moderate intensity	Brisk walking, treadmill	Increased CBF, frontal GM preservation	Executive function and cognitive flexibility	Provides the broadest overall cognitive benefits, combining vascular and structural adaptations.
High intensity	Vigorous aerobic training, cycling progression	Cortical thickness, network efficiency	Executive control, sensory processing	Larger structural adaptations, especially in at-risk adults
Dual-Task	Aerobic exercise + cognitive training, exergames	Greater network integration and neural efficiency	Inhibition, flexibility, working memory	Synergistic effects beyond exercise alone
Aerobic + sensory enrichment	Aerobic exercise with music and dancing	Frontal preservation, multisensory activation	Visuospatial function, divided attention, gait	Additional benefits for motor-cognitive integration

While aerobic exercise alone improves cognition, combining it with cognitive training appears to amplify these benefits, especially in domains like executive control (41, 55–59). However, the promise of dual interventions improvement, must be tempered by a critical examination of what “added value” means. The additive and synergistic effects reported across studies are typically defined relative to performance on the same executive function tasks used during or immediately after training, a narrow benchmark that does not speak to broader cognitive transfer. The claim that dual interventions “better mimic real-life cognitive demands” is theoretically appealing but empirically unverified: no study reviewed here directly compared dual intervention outcomes against ecologically valid decision-making measures. This caution is consistent with the broader meta-analytic literature; cognitive training generally produces small gains on trained or closely related tasks; exercise interventions similarly yield only modest improvements on executive-function measures, with weaker evidence when compared against active control conditions (60, 61). These patterns indicate that superior performance following dual interventions cannot, by itself, establish either broad transfer between their components. Furthermore, the mechanisms proposed to explain synergistic effects, simultaneous cardiovascular and cognitive engagement, cross-domain neural activation, enhanced arousal, remain largely inferential. It is unclear, for instance, whether the greater PFC/ACC activation observed following dual interventions reflects genuine capacity expansion, more efficient neural recruitment, or simply greater task engagement during assessment. Resolving this ambiguity will require intervention designs that move beyond standard neuropsychological batteries and incorporate decision-making paradigms that capture more naturalistic environment, and follow-ups to assess the duration and generalization of the results to participants' real life.

Furthermore, individual outcomes remain highly variable. Factors such as age, baseline cognitive status, fitness level, and genetics likely influence responsiveness. Moreover, training type matters: aerobic exercise tends to enhance executive function and PFC activation, while resistance training may better support memory and processing speed (33, 62–64). Mind-body practices like Tai Chi and yoga also offer cognitive benefits, combining moderate aerobic effort with attentional and sensorimotor engagement (39, 48, 50, 65–71). Importantly, acute vs. chronic interventions differ in effect: while a single bout of mild exercise may produce short-lived cognitive boosts, sustained programs foster longer-term neuroplasticity. Virtual reality and dual-task training further support the value of simultaneous cognitive and physical stimulation (72, 73). Ultimately, a one-size-fits-all model is insufficient.

### 4.1 Transfer effects: a path to closing the gap

Although physical and combined interventions showed improved performance on specific EF tasks, broader transfer to untrained domains or real-world outcomes remains limited to determine. Transfer is classified as near, when improvements occur in tasks structurally similar to those used in training; far, when gains are observed in distinct but related cognitive domains; and ecological, when benefits generalize to real-world functioning (74). Despite the central role of executive functions in daily life (75), none of the studies included in this review explicitly assessed any type of transfer effect. This raises important questions about how to interpret the functional significance of cognitive gains and neural changes, as well as the mechanisms that support their generalization.

Several methodological and conceptual factors constrain the detection of transfer. For instance, many studies relied on pretest-posttest designs using identical or highly similar instruments, which are more likely to capture practice effects than generalizable changes. Additionally, tasks that appear behaviorally similar may rely on distinct cognitive operations, reducing the likelihood of functional overlap (76). Furthermore, the instruments used were originally developed for diagnostic purposes and administered under controlled conditions, which may limit their sensitivity to detect subtle changes in healthy adults and fail to capture motivational or sociocultural variables relevant to everyday functioning (77).

To address these challenges, the capacity-efficiency framework might contribute to elucidating the extent of the effects of different kinds of intervention. It distinguishes between improvements resulting from expanded cognitive resources (capacity) and those arising from more effective deployment of existing resources (efficiency) (74). Although reduced frontoparietal activation is compatible with increased efficiency (78), neural recruitment is not uniquely interpretable: greater bilateral activation may provide no behavioral benefit or additional task-relevant information and may instead reflect inefficiency, dedifferentiation, or reduced interhemispheric inhibition (75, 79). If exercise-induced cognitive gains, their benefits may be most evident under low-to-moderate cognitive demands, where improved processing strategies can support performance without exceeding current capacity limits (78). By contrast, high load conditions may expose residual capacities constraints once compensatory recruitment reaches its functional ceiling (80).

Future research can address these challenges by designing interventions aimed to generalize cognitive gains to everyday functioning. We propose organizing this framework around seven methodological domains. First, study populations should be characterized multidimensionally, and the term healthy should be defined operationally rather than inferred as absence of clinical diagnoses or performance above a cognitive-screening threshold. Second, intervention components, dose, progression, adherence, and implementation fidelity should be reported in sufficient detail for replication and interpretation. Factorial designs should be preferred when combined using interventions to isolate component-specific, additive, or synergistic effects. Third, control conditions should be selected according to the causal effect being estimated and described with the same rigor as the experimental intervention. Active controls provide more specific estimates by accounting for expectancy, social contact, supervision, repeated assessment, behavioral activation, or general cognitive and physical engagement. Their content, dose, expected benefits, social exposure, temporal demands, adherence, and changes in participant's activities outside the study should be documented (65, 75, 81). Fourth, primary outcomes and transfer levels should be prespecified. Multiple, sufficiently independent tasks should be used to represent each target construct. Fifth, repeated assessments should be addressed through alternate forms, estimates of test-retest effects, appropriate comparators and longitudinal measurement invariance. Statistical significance should be distinguished from reliable individual change, clinical relevance, and real-world benefit. Sixth, neural findings should be integrated with behavioral and physiological measures rather than interpreted independently. Compensation, efficiency, capacity expansion, and qualitative reorganization should be treated as competing or complementary hypotheses. Finally, follow-up assessments and prespecified real world outcomes are necessary to distinguish immediate task learning from maintained cognitive change, transfer to everyday activities, and modification of longer-term aging trajectories.

In conclusion, the available evidence supports a constrained but meaningful conclusion. Physical and cognitive interventions can modify performance in later adulthood, demonstrating that aging cognitive executive systems remain plastic. The most reliable effects concern trained tasks and, to a lesser degree, closely related measures. Effects on broader executive constructs are generally small and heterogeneous, far transfer is not consistently supported, and ecological or clinical benefits remain insufficiently demonstrated. The field should therefore move away from the binary question of whether an intervention “works” and toward a layered evaluation of what changes, for whom, through which mechanism, under which comparison, for how long, and in what context. A change in a cognitive score should be treated as the beginning of this analysis rather than its endpoint. Only convergent evidence of measurements, equivalence, causal attribution, mechanism, persistence, and functional generalization can justify claims that a physical, cognitive or dual intervention strengthens executive functioning or meaningfully alters aging.
